# Relative Metabolic Stability, but Disrupted Circadian Cortisol Secretion during the Fasting Month of Ramadan

**DOI:** 10.1371/journal.pone.0060917

**Published:** 2013-04-18

**Authors:** Suhad Bahijri, Anwar Borai, Ghada Ajabnoor, Altaf Abdul Khaliq, Ibrahim AlQassas, Dhafer Al-Shehri, George Chrousos

**Affiliations:** 1 Department of ClinicalBiochemistry–Faculty of Medicine, King Abdulaziz University, Jeddah, Saudi Arabia; 2 Saudi Diabetes Study Research Group, King Fahd Medical Research Center, King Abdulaziz University, Jeddah, Saudi Arabia; 3 King Abdullah International Medical Research Center, King Abdulaziz Medical City, Jeddah, Saudi Arabia; 4 Department of ClinicalBiochemistry, Faculty of Allied Medical Sciences, Um Alqura University, Makkah, Saudi Arabia; 5 Department of Pediatrics, University of Athens Medical School, “Aghia Sophia” Children's Hospital, Athens, Greece; University of Warwick – Medical School, United Kingdom

## Abstract

**Background:**

Chronic feeding and sleep schedule disturbances are stressors that exert damaging effects on the organism. Practicing Muslims in Saudi Arabia go through strict Ramadan fasting from dawn till sunset for one month yearly. Modern era Ramadan practices in Saudi Arabia are associated with disturbed feeding and sleep patterns, namely abstaining from food and water and increasing daytime sleep, and staying awake and receiving food and water till dawn.

**Hypothesis:**

Strict Ramadan practices in Saudi Arabia may influence metabolism, sleep and circadian cortisol secretion.

**Protocol:**

Young, male Ramadan practitioners were evaluated before and two weeks into the Ramadan. Blood samples were collected at 9.00 am and 9.00 pm for measurements of metabolic parameters and cortisol. Saliva was collected serially during the day for cortisol determinations.

**Results:**

Ramadan practitioners had relative metabolic stability or changes expected by the pattern of feeding. However, the cortisol circadian rhythm was abolished and circulating insulin levels and HOMA index were increased during this period.

**Discussion:**

The flattening of the cortisol rhythm is typical of conditions associated with chronic stress or endogenous hypercortisolism and associated with insulin resistance.

**Conclusions:**

Modern Ramadan practices in Saudi Arabia are associated with evening hypercortisolism and increased insulin resistance. These changes might contribute to the high prevalence of chronic stress-related conditions, such as central obesity, hypertension, metabolic syndrome and diabetes mellitus type 2, and their cardiovascular sequelae observed in the Kingdom.

## Introduction

Disruption of feeding and sleep schedules have adverse effects on affect and metabolism [1], [2], [3], [4]. Both conditions are stressors that influence the stress system and the secretion of its mediators, including corticotropin-releasing hormone, the catecholamines norepinephrine and epinephrine and cortisol, through which they exert damaging effects [5]. The chronicity of these stressors is key, as stress hormones are meant to act as homeostatic mediators in a time-limited fashion, allowing tissue repair and functionality once they no longer exist [6].

The Muslims represent roughly 1/4^th^ of humanity [7]. The religious fasting of the Ramadan takes place once a year and lasts one month, i.e. accounts for about 8 percent of a practicing adult Muslim's life. During Ramadan, there is no food or water taken up from dawn to sunset. Instead, all feeding and water uptake take place from sunset to dawn. Traditional fasting practices in the past did not affect daily routine dramatically. However with modernization and availability of electricity, lifestyle changed dramatically, especially in the gulf countries. Staying up till dawn became a common practice among all age groups and socioeconomic classes, curtailing the duration of sleep and disturbing its quality. These stressful changes during the Ramadan are accentuated when it takes place in the summer, when the daylight hours are increased.

This study examined the effect of Ramadan fasting and disturbance of sleep patternson markers of metabolism and circadian cortisol secretion of practicing healthy young Saudi Arabian men and women.

## Methods

### Subjects and study design

Studying the same subjects at different times helps to avoid variability between groups at base time, and allows for smaller sample number. Based on laboratory quality control data, and reference ranges for intended measurements, sample size was calculated to avoid type II statistical error [8]. Since serum cortisol showed the greatest variability, and its analytical method had the highest coefficient of variation, a difference of 20% in its mean was adopted to calculate the required sample size, which was found to be 18.7. This was increased further to account for drop-outs, and the total number of recruited subjects was 25. The protocol was approved by the Committee on the Ethics of Human Research at the “Faculty of Medicine- King Abdulaziz University”. Twenty-fourvolunteer healthy subjects (19 males, 5 females), aged 18–42 years, were recruited and completedthe study. Written informed consent was obtained in all cases. Volunteers were studied twice, during their regular life before, and again 10–15 days into the Ramadan (fasting) period. They were instructed to have meals as usual on the day of testing, and to record their usual sleeping and waking times for the previous three days. Anthropometric and blood pressure measurements were obtained by a team of two trained observers using calibrated tools.

**Figure 1:**
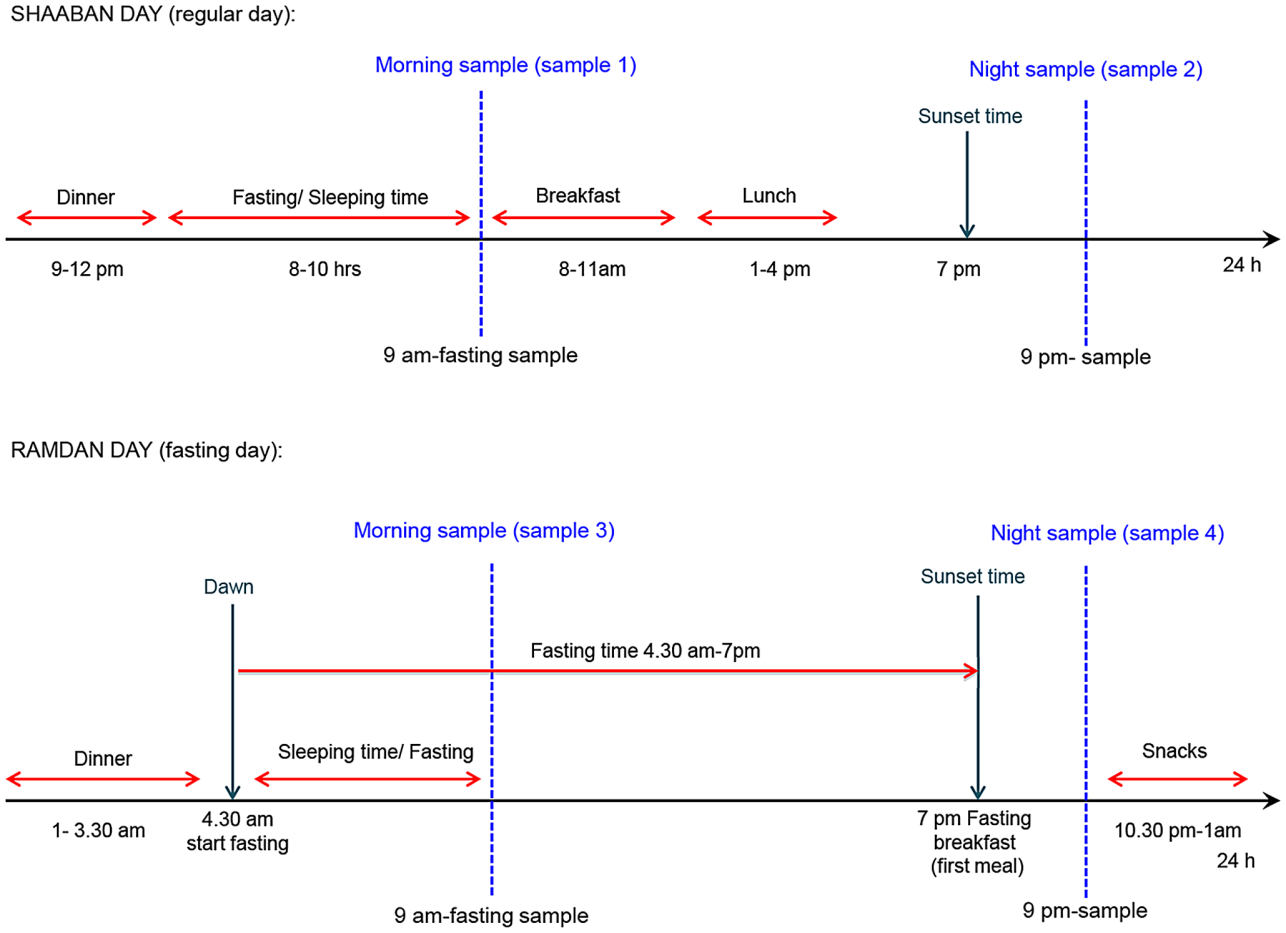
Meal time and sleep patterns during the pre-fasting month of Shaaban and the fasting month of Ramadan.

Blood samples were drawn twice daily at 9 am (±1 hour) and again twelve hours later. Thus, sample one and three were obtained while fasting (at least 10 hours for sample one and 6–7 hours for sample 3), and samples two and four were obtained 2–5 hours after meals as shown in Figure 1.

The subjects were also given salivettes (SARSTEDT) to collect saliva every 4 hours for 24 hours, except when asleep, during both days of blood sampling. Salivary samples were collected using the salivette tubes and cotton swabs saturated with citric acid (SARSTEDT, Cat. 51.1534.001). Salivettes are specially designed for cortisol determination in saliva (recovery ∼100% after swab centrifugation). Salivettes were stored in the refrigerator during collection time, and brought to the laboratory for storage in the next morning. Separated serum samples, as well as centrifuged saliva samples, were stored at −80°C until measurements were performed.

### Biochemical and endocrine assays

Serum biochemical and endocrine parameters were assayed in the accredited Clinical Chemistry laboratories of the “National Guard Hospital” in Jeddah. Serum glucose and lipids (cholesterol, triglycerides and high density lipoprotein (HDL-C) were assayed on ABBOTT Architect c16000 auto-analyzer, which was also employed for the quantitative determination of electrolytes (Sodium, Potassium and Chloride) in serum using the principle of Integrated Chip Technology (ICT). Low density lipoprotein- cholesterol (LDL-C) was calculated using the Friedewald equation [9]. Insulin was measured by a chemiluminescent microparticle immunoassay (CMIA) on ABBOTT Architect i1000 auto-analyzer. Insulin and glucose values were used to calculate homeostasis model assessment (HOMA-IR) insulin resistance equation [10]. Serum cortisol was determined by achemiluminescence immunoassay using Immulite1000. All tests were kindly donated by Abbott Medi-Serve (Saudi Arabia).

Salivary cortisol was assayed at the “Nutrition Research Unit” at King Fahd Medical Research Centre by IBL-AMERICA Salivary Cortisol HSELISA kit using Thermo Labsystems Ultra Wash Plus microplate washer and Multilabel Counter VICTOR model 1420 plate reader from PerkinElmer, UK. The assay is a solid phase enzyme-linked immunosorbent assay (ELISA), based on the principle of competitive binding with a maximum intra- and inter-assay coefficient of variation (CV) of 9.1 % and 11.9%, respectively, and analytical sensitivity of 0.012 ng/mL.

### Statistical analysis

Analyses were performed using SPSS statistical package version 16. Descriptive statistics, such as mean ± SEM, were calculated for all estimated parameters. Paired Student t-test, and the Mann Whitney-U test were employed for comparison of normally distributed and non-normally distributed parameters, respectively. Linear regression analysis was carried out for Shaaban and Ramadan separately with salivary cortisol concentrations being the dependent variables and time being the independent variable. Significance was assigned at p<0.05.

## Results

One male volunteer dropped out of the study leaving 23 subjects (18males and 5 females). Subjects slept for 7–10 hours daily during their regular (non- fasting) life, divided between a night sleep of 6–8 hours and an afternoon nap for 1–2 hours. During Ramadan, they stayed up till sunrise (5∶00 am), mostly sleeping for 4–6 hours, depending on working conditions, then again in the afternoon for 1–3 hours. Some napped after breaking their fast between 7∶30 pm and 8∶30 pm. Thus, the total number of hours/day during Ramadan was 6–7 hours of non-consolidated sleep, mostly during the daytime. Demographic and anthropometric characteristics of the group are summarized in Table 1. Twenty-two subjects were <30 years of age. 16 subjects had a normal BMI (<25 Kg/m^2^), 4 were overweight (25–<30 Kg/m^2^) and 3 obese (≥30 Kg/m^2^). One male subject with normal BMI had undiagnosed elevated blood pressure (154/94). Results of estimated biochemical and endocrine parameters are presented in Table 2.

**Table 1:** Demographic and anthropometric characteristics of the study group at the first visit.

	Mean ± SEM	Min.	Max.
Age (yrs)	23.1±1.2	18.0	42.0
Weight (Kg)	70.9±3.6	46.1	118.9
Height (cm)	169.5±1.8	152.0	180.0
Waist circumf. (cm)	85.9±2.9	67.0	123.0
Body fat (%)	25.2±1.9	10.7	48.5
BMI (Kg/M^2^)	24.6±1.1	18.6	41.1
Systolic BP	125±3	108	154
Diastolic BP	79±2	66	94
Hours of sleep/Day	7.3±0.3	6.0	10.0

BP, Blood pressure.

**Table 2:** Biochemical and endocrine parameters in the morning and evening samples collected on the two visits.

		Shaaban		Ramadan		Shaaban-Ramadan
		Mean ± SEM	P value (AM-PM)	Mean ± SEM	P value (AM-PM)	P value
Glucose (mmol/L)	AM	5.33±0.07	0.28	5.62±0.11	0.38	0.01
	PM	5.48±0.14		5.46±0.17		0.94
Insulin (µU/ml)	AM	8.25±0.98	0.00	16.86±3.36	0.00	0.01
	PM	19.59±2.90		50.17±5.74		0.00
Total Cholesterol (mmol/L)	AM	4.52±0.19	0.35	4.51±0.15	0.27	0.97
	PM	4.56±0.20		4.70±0.17		0.15
Triglycerides (mmol/L)	AM	0.85±0.12	0.00	1.21±0.11	0.96	0.00
	PM	1.20±0.13		1.36±0.15		0.28
HDL-Cholesterol (mmol/L)	AM	1.20±0.05	0.66	1.10±0.03	0.47	0.00
	PM	1.21±0.05		1.14±0.04		0.01
LDL-Cholesterol (mmol/L)	AM	2.93±0.16	0.42	2.86±0.13	0.12	0.51
	PM	2.80±0.17		3.00±0.15		0.09
Sodium (mmol/L)	AM	138.7±0.26	0.79	138.1±0.27	0.27	0.25
	PM	138.0±0.29		139.3±0.43		0.06
Potassium (mmol/L)	AM	4.03±0.053	0.08	3.96±0.051	0.61	0.27
	PM	3.91±0.055		3.93±0.047		0.75
Chloride (mmol/L)	AM	104.1±0.400	0.33	104.3±0.403	0.75	0.8
	PM	105.0±0.405		104.4±0.319		0.10
Cortisol (nmol/L)	AM	326.4±28.1	0.00	247.9±30.1	0.07	0.06
	PM	193.9±29.1		251.7±22.5		0.01
Cortisol Ratio (AM/PM)	–	2.55±0.38	–	1.22±0.20	–	0.00
HOMA- IR	–	1.98±0.24	–	4.51±1.04	–	0.01

### Electrolytes

The levels of sodium, potassium and chloride were within the normal range and remained stable throughout the study, with no statistical differences between morning and evening levels, nor between levels in samples taken during the non-fasting or the fasting month (p>0.05 in all cases) (Table 2).

### Glucose and Insulin

No values outside the normal range were noted for glucose. Glucose level was kept stable throughout the day during the two visits as seen from the p value obtained by comparing fasting with evening values (p>0.05 on both occasions). However, as expected, fasting level during Ramadan was significantly higher than the level obtained during the first visit due to the shorter length of time after the last meal, as seen in Table 2. On the other hand, insulin level was significantly increased in the evening on both visits (see Table 2 for p values). Moreover, morning and evening insulinlevels in Ramadan were significantly higher than corresponding ones in Shaaban (p = 0.014 and <0.001 respectively). Thisincrease was reflected on significantly higher HOMA-IR value (p = 0.023) during themonth of Ramadan.

### Lipids

All subjects had normal lipids profile during the first visit. Similar to was noted for glucose level, there was no statistically significant difference between morning and evening levels for total cholesterol, and HDL – or LDL-cholesterol during the two visits (p>0.05 in all cases). However, HDL – cholesterol showed a statistically significant decrease during the fasting month (p<0.001 and 0.011 for morning and evening samples, respectively).

The evening samples had higher levels of triglycerides compared to fasting samples, but only significantly so during the non-fasting month (p = 0.003). In addition, the level in fasting samples during Ramadan was significantly higher than corresponding ones during the non-fasting month.

### Serum Cortisol

Cortisol level was significantly higher in the morning than in the evening during the non-fasting month (p = 0.001). However, this difference was abolished during Ramadan (p = 0.068). Morning level was lower in Ramadan compared with the non-fasting month, but not significantly so (p = 0.06). Moreover, the evening cortisolin Ramadan was significantly higher than during the non-fasting month (p = 0.008). This was reflected in a significantly lower AM/PM cortisol ratio during Ramadan (p = 0.004) (Table 2).

### Salivary Cortisol

**Figure 2:**
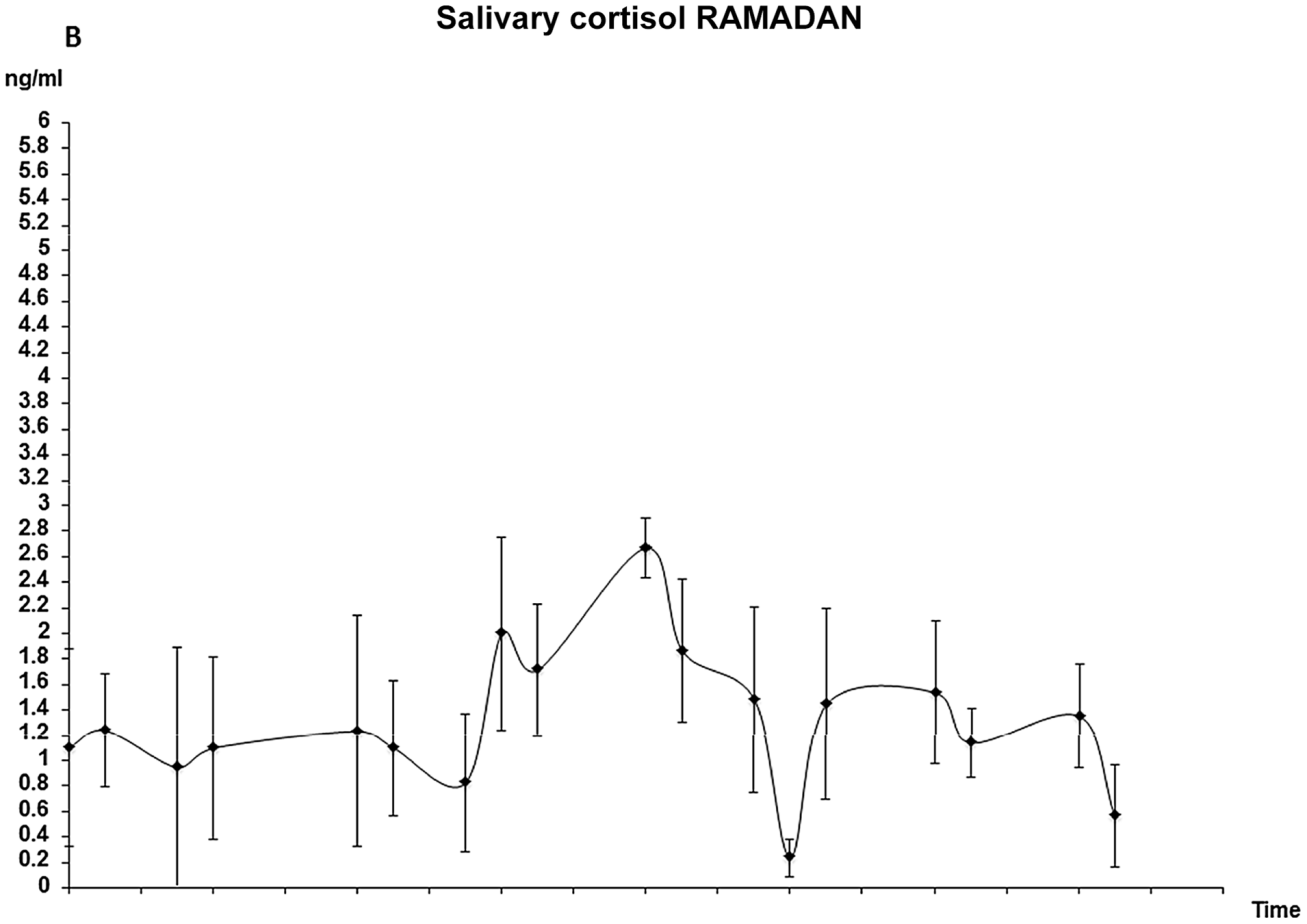
Morning and evening values of salivary cortisol during a) the non-fasting and b) the fasting month.

Due to different social, working and sleeping schedules, samples were collected at different times of the day. Results through waking hours of Shaaban and Ramadan are presented Figure 2 a) and b). Each point on the figure represents an average of ≥6 samples ± SD. In confirmation ofthe serum cortisol results, salivary cortisol values showed an obvious flattening of circadian cortisol secretion during the fasting monthcompared to the non-fasting month; with lower levels during mid-morning and higher levels in the evening and early morning. This observation was confirmed by results from linear regression analyses, which indicated significantly decreased values in the evening during the non-fasting month (p = 0.018), but no such a decrease during the fasting month of Ramadan (p = 0.254).

## Discussion

Our study is the first to study the pattern of daily cortisol secretion in healthy Saudi subjects before and during the fasting month of Ramadan, and try to relate changes in eating and sleeping patterns to changes in metabolic and endocrine markers.

The circadian pattern of circulating cortisol was disrupted during the Ramadan fasting, as levels were lower in the morning and quite high in the evening compared to the pre-Ramadan values. This flattening of the circadian cortisol curve has been seen before in patients with chronic stress [11], [12], chronic anxiety disorder [13], [14], melancholic depression [15], night shift work [16], [17] and endogenous Cushing syndrome [3], [18], all chronic conditions characterized by metabolic disturbances typical of the (dys) metabolic syndrome [11], [19]. In spite of this, most of the metabolic parameters examined, such as serum glucose, cholesterol, triglycerides, LDL- and HDL-cholesterol, presented with remarkable stability or explained by the feeding pattern of the subjects. Insulin elevations in the evening were able to maintain glucose levels in the normal range, while serum electrolytes were quite stable.

Higher evening cortisol during the month of Ramadan was also reported in earlier studies [20], [21], with the first one carried out in Saudi Arabia on a much smaller number of subjects. However, no attempt was made to study the effect on markers of metabolism, or to follow the daily secretion rhythm.

The glucocorticoid receptor (GR) is tightly linked to the biological clock [22], [23]. The Clock/BMAL1 heterodimer transcription factor, whose activity oscillates in a circadian fashion with peak activity in the morning, when cortisol levels are normally at their peak, interacts directly with tissue GR throughout the body and acetylates it [24], [25]. The acetylated form of the receptor is less active than the native one [26]. In the evening, through the action of deacetylases, the receptor regains its transcriptional activity and the tissues become more sensitive to glucocorticoids, with peak sensitivity at the time of the least Clock/BMAL1 activity [27], [28]. Therefore, the noted elevations of cortisol concentrations in the evening are particularly worrisome as they occur at the time of maximum tissue sensitivity to glucocorticoids. This might explain the noted increased HOMA-IR (reflecting increased insulin resistance) during Ramadan.

In addition to the reversal of feeding schedule and the disruption this causes in metabolism, practicing subjects have decreased duration and impaired quality of sleep as noted earlier. Whereas sleep loss is a stressor in its own right, cortisol levels following a night of curtailed or poor quality sleep are not usually elevated, probably as a result of sleep center-dependent inhibition of the stress system including the hypothalamic-pituitary-adrenal axis [26], [27], [28], [29], [30]. Yet, sleep deprivation over time leads to elevation of somnolence-inducing proinflammatory cytokines, such as Interleukin-6, which however exert untoward metabolic effects [30], including insulin resistance [31].

Our findings suggest that during the month of strict Ramadan fasting accompanied by a greatly disturbed sleeping pattern, as it occurs in Saudi Arabia and probably other Muslim countries as well, may be associated with the hypercortisolism of chronic stress. The high levels of cortisol in the evening, at the time of maximum glucocorticoid sensitivity of peripheral tissues, might, over many years, contribute to the increased prevalence of chronic stress-related conditions, such as central obesity, hypertension, metabolic syndrome and diabetes mellitus type 2, and their cardiovascular sequelae observed in Saudi Arab citizens as suggested in some studies [31].

Modern life, with the advent of artificial light and massive nutrient production, has curtailed sleep duration and increased availability of low quality food. This is quite different from pre-modern life, when sleep onset was much earlier in the evening and sleep duration at least 2 hours longer. What religious tradition dictated under a more natural setting over a thousand years ago should probably be adjusted to today's environment to minimize the negative effects of the stress of fasting and sleep deprivation on the body.
