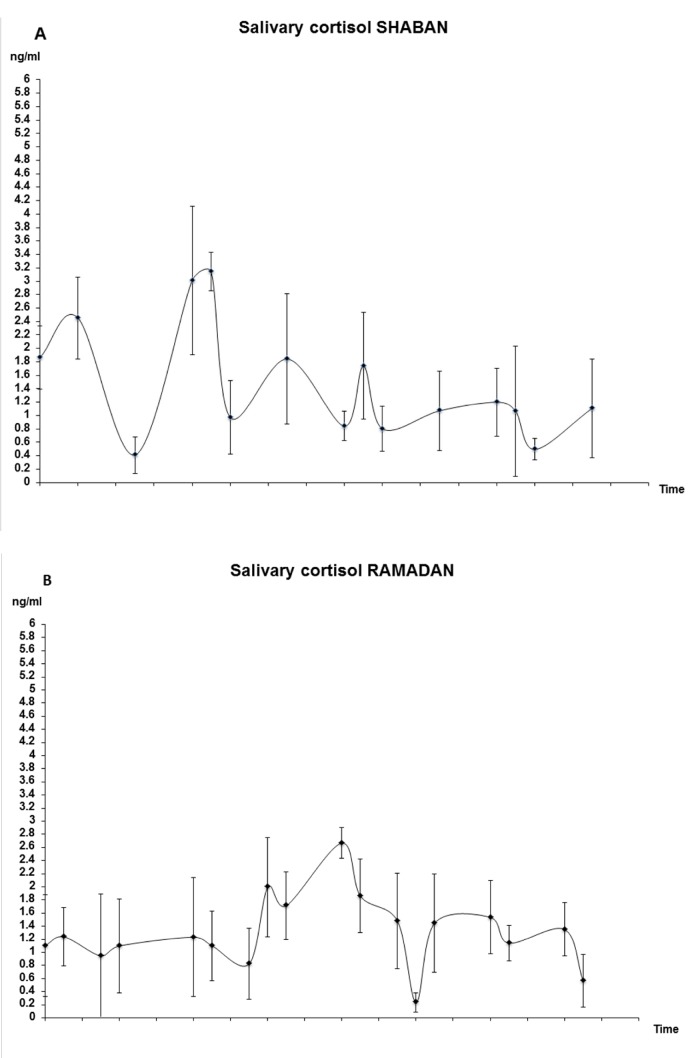# Correction: Relative Metabolic Stability, but Disrupted Circadian Cortisol Secretion during the Fasting Month of Ramadan

**DOI:** 10.1371/annotation/8d92315c-9944-4470-9bbb-806a26b0809b

**Published:** 2013-06-19

**Authors:** Suhad Bahijri, Anwar Borai, Ghada Ajabnoor, Altaf Abdul Khaliq, Ibrahim AlQassas, Dhafer Al-Shehri, George Chrousos

Panel A is missing from Figure 2. Please see the correct version of Figure 2 at the following link:

**Figure:**